# The Administration of Anæsthetics

**Published:** 1907-10-19

**Authors:** 


### HOSPITAL ADMINISTRATION.

### CONSTRUCTION AND ECONOMICS.

### THE ADMINISTRATION OF ANÆSTHETICS.

### SHOULD HOUSE OFFICERS BE ALLOWED TO GIVE ANÆSTHETICS?

Once again there has been a black week of anæsthetic fatalities, though the major calamities of railway and tramcar hecatombs have perhaps overshadowed them and diverted the popular appetite for sensation elsewhere. For the profession, however, the inquests concluded since the last issue of this journal raise points of the greatest importance. These points have, it is true, been raised before, and discussed in these columns on June 8, July 6 and July 20 of this year. But while the settlement of them is still *sub judice*, we cannot but admit every fresh piece of evidence which bears upon the case. In our previous comments we dwelt with emphasis upon some of the difficulties which beset the exhibition of anæsthetic drugs to the moribund subjects of strangulated hernia and similar conditions which are by common consent included under the term "emergency operations." And we especially drew attention to the fallacy of comparing mortality statistics without sifting them very carefully.

Unfortunately, contributory and unavoidable circumstances of this nature cannot, if newspaper reports are to be relied upon, be advanced in explanation of the latest fatalities. Of the three cases to which we refer, two occurred in Guy's Hospital and one in St. Bartholomew's. Consequently the investigation of all three has fallen to the lot of Dr. Waldo, Coroner for the City and for Southwark, who has taken pains in all these cases for many years, and whose outspoken comments carry weight with the Press as well as with the profession. Between the two cases at Guy's there is a good deal of similarity. In each the A.C.E. mixture was administered, and in each this was done by a house officer. The terms of office served by these gentlemen seem to have been three months and three days respectively, so that, without reflecting in any way upon their skill and ability, the Coroner was amply justified in remarking on their lack of experience. According to the published reports, one of the administrators deposed to a total experience of ninety-eight anæsthetisations, including a "fair number" of chloroform cases; the other had officiated 120 times since his admission to the register. It would be of interest to know how many of these were under expert supervision. In both cases, moreover, death occurred during or immediately after the induction period of anæsthesia; in short, during the most dangerous part of a chloroformisation. In both cases the patients were suffering from morbid states closely affecting or involving the air passages. One case was that of a woman suffering from exophthalmic goitre; the other that of a self-inflicted cut throat in which the stitched wound was being reopened for drainage. It is therefore relevant and pertinent to inquire, as Dr. Waldo has done, why these two cases, neither of them "emergencies" in the hospital sense, and both of them calling for especially skilled anæsthetisation, should have been entrusted to house officers of extremely limited experience, rather than to one of the eight visiting anæsthetists of whom mention is always made at these inquests. The only answer to be gathered from the accounts, and we feel sure it must be incorrectly reported, is the statement from the witness-box attributed to one of the house officers concerned, that of those gentlemen "none attended on Wednesday."

In the summing-up of the goitre case, after allusion to the evidence of the operating surgeon as to the special anæsthetic difficulties of this class of case, and to that of the house surgeon, who admitted never having administered to a thyroid case before, the question of the system in force at Guy's is discussed in forcible terms. The Coroner said to the jury: "In an operation of the kind under investigation there was no great urgency, so that the services of the visiting anæsthetist could readily have been secured. Under these circumstances, gentlemen, it becomes your serious duty to consider whether it was right to entrust the chloroform, in the case of the deceased, to a house surgeon whose experience has been confined to 98 cases of administration of anæsthetics. The question thus raised is one of system. . . . We all recognise the necessity of medical men being properly instructed in the administration of anæsthetics, but I think most of us will also agree that all cases of admitted difficulty, as, for instance, those of operation about the throat or upper air passages, should be entrusted only to anæsthetists of long experience. That appears to be recognised by the appointment of eight visiting anæsthetists at Guy's."

To these strictures, on the evidence produced, it is difficult to withhold assent: for the credit of their hospital and of the medical profession, as well as for the protection of their house officers from a certain amount of unpleasant public notoriety, *the authorities at Guy's would do well to devise some improvements on a system which appears to work uncommonly ill*, if it is true, as we gather from Dr. Waldo's figures, that *eleven fatalities during anæsthesia have occurred in that hospital in the last six months*. Either the teaching and training of students, or the system by which anæsthetists are selected from among the house officers, is radically at fault. Other hospitals do not show records such as this, and we cannot forget the two fatalities earlier in the year, which happened while newly qualified men holding no appointment whatever were permitted to give anæsthetics in the hospital without supervision. We say this deliberately, because we agree that Dr. Waldo's remarks are an inevitable commentary on the inquests he so frequently holds. On the broad question of the administration of anæsthetics by house officers we do not entirely agree with him, as explained in detail on p. 259, vol. i., but there is no shirking the fact that his deductions are certainly justified by his experiences in the Borough. *However high the reputation of Guy's Hospital in the district which it serves, it cannot indefinitely survive a series of such calamities when they occur once a fortnight.*

We turn next to the third case which Dr. Waldo has been investigating. This was at St. Bartholomew's Hospital, and was that of a man who received a severe blow on the head from a brick, and died after inhaling one and a-half drachms of chloroform given for the purpose of enabling a house surgeon to reach some bleeding vessel. In this case the administrator is one of the regular anæsthetic staff, as has been the case with all the fatalities from this hospital in recent years. One reason of this is, no doubt, the much larger proportion of administrations by the staff at this hospital, but this is probably not by any means the whole explanation, for certainly a great number of anæsthetics are administered there yearly by the residents, without the lamentable incidents which follow that practice at Guy's. This raises the old question as to the purity and efficiency of the drugs used: at Guy's the cheaper acetone chloroform and methylated ether are used, whereas at St. Bartholomew's the costly products of ethyl alcohol are in favour. The statistics of other hospitals do not favour any invidious comparisons between the two forms of the drugs, so there seems little to fall back on other than some dissimilarity in the system and teaching at the two hospitals. It is, at any rate, interesting to note how once again the induction period has proved to be the fatal one, as is so often the case with chloroform or any mixture containing it.

